# Supplementary material for: Nanoapatites Doped and Co-Doped with Noble Metal Ions as Modern Antibiofilm Materials for Biomedical Applications against Drug-Resistant Clinical Strains of Enterococcus faecalis VRE and Staphylococcus aureus MRSA
Source: Int J Mol Sci. 2022 Jan 28;23(3):1533. doi: 10.3390/ijms23031533 (PMC8836119; doi:10.3390/ijms23031533)
Supplement: Supplementary file 1 [file ijms-23-01533-s001.zip › ijms-1557351-supplementary.pdf]

International Journal of *Molecular Sciences*

MDPI

Supplementary material

Nanoapatites doped and co-doped with noble metal ions as modern antibiofilm materials for biomedical applications against drug-resistant clinical strains of *Enterococcus faecalis VRE* and *Staphylococcus aureus* MRSA

Emil Paluch [1,*], Paulina Sobierajska [2,*], Piotr Okinczyc [3], Jaroslaw Widelski [4], Anna Duda-Madej [1], Barbara Krzyzanowska [1], Paweł Krzyzek [1], Rafal Ogorek [5], Jakub Szperlik [6], Jacek Chmielowiec [2], Grazyna Gosciniak [1] and Rafal J. Wiglusz [2,*]

[1] Department of Microbiology, Faculty of Medicine, Wroclaw Medical University, 50-376 Wroclaw, Poland; emil.paluch@umw.edu.pl (E.P.); anna.duda-madej@umw.edu.pl (A.D-M.); pawel.krzyzek@umw.edu.pl (P.K.); barbara.krzyzanowska@umw.edu.pl (B.K.); grazyna.gosciniak@umw.edu.pl (G.G.)

[2] Institute of Low Temperature and Structure Research, Polish Academy of Sciences, Okolna 2, 50-422 Wroclaw, Poland; p.sobierajska@intibs.pl (P.S.); j.chmielowiec@intibs.pl (J. C.); r.wiglusz@intibs.pl (R. J. W.)

[3] Department of Pharmacognosy and Herbal Medicines, Wroclaw Medical University, 50-556 Wroclaw, Poland; piotr.okinczyc@umw.edu.pl (P.O.)

[4] Department of Pharmacognosy with the Medicinal Plant Garden, Medical University of Lublin, 20-093 Lublin, Poland; jaroslaw.widelski@umlub.pl (J.W.)

[5] Department of Mycology and Genetics, University of Wroclaw, Przybyszewskiego 63, 51-148 Wroclaw, Poland; rafal.ogorek@uwr.edu.pl (R.O.)

[6] Faculty of Biological Sciences, Botanical Garden, University of Wroclaw, Sienkiewicza 23, 50-525 Wroclaw, Poland; 11 jakubsz@hotmail.com (J.S.)

* Correspondence: emil.paluch@umw.edu.pl (E.P.); p.sobierajska@intibs.pl (P.S.), r.wiglusz@intibs.pl (R.J.W.)

Table of content:

Results:

Figure S1. *EDS spectra of the nHAp and nanoapatites doped, double-doped and triple-doped with Ag^+, Au^+ and Pd^{2+} ions.*…………………..s2

Figure S2. *Representative SEM images of the nHAp pellet and nanoapatites pellets doped with Ag^+, Au^+ and Pd^{2+} ions* ……………….……. s3

Figure S3. *Adhesion of Balb/3T3 fibroblasts to surface tested nanoapatites and influence of the biofilm produced by E. faecalis VRE 200 and S. aureus MRSA P19 on the viability of Balb/3T3 fibroblasts………..…………...………s4*

Results

EDS spectra of the nHAp and nanoapatites doped, double-doped and triple-doped with Ag^{+}, Au^{+} and Pd^{2+} ions

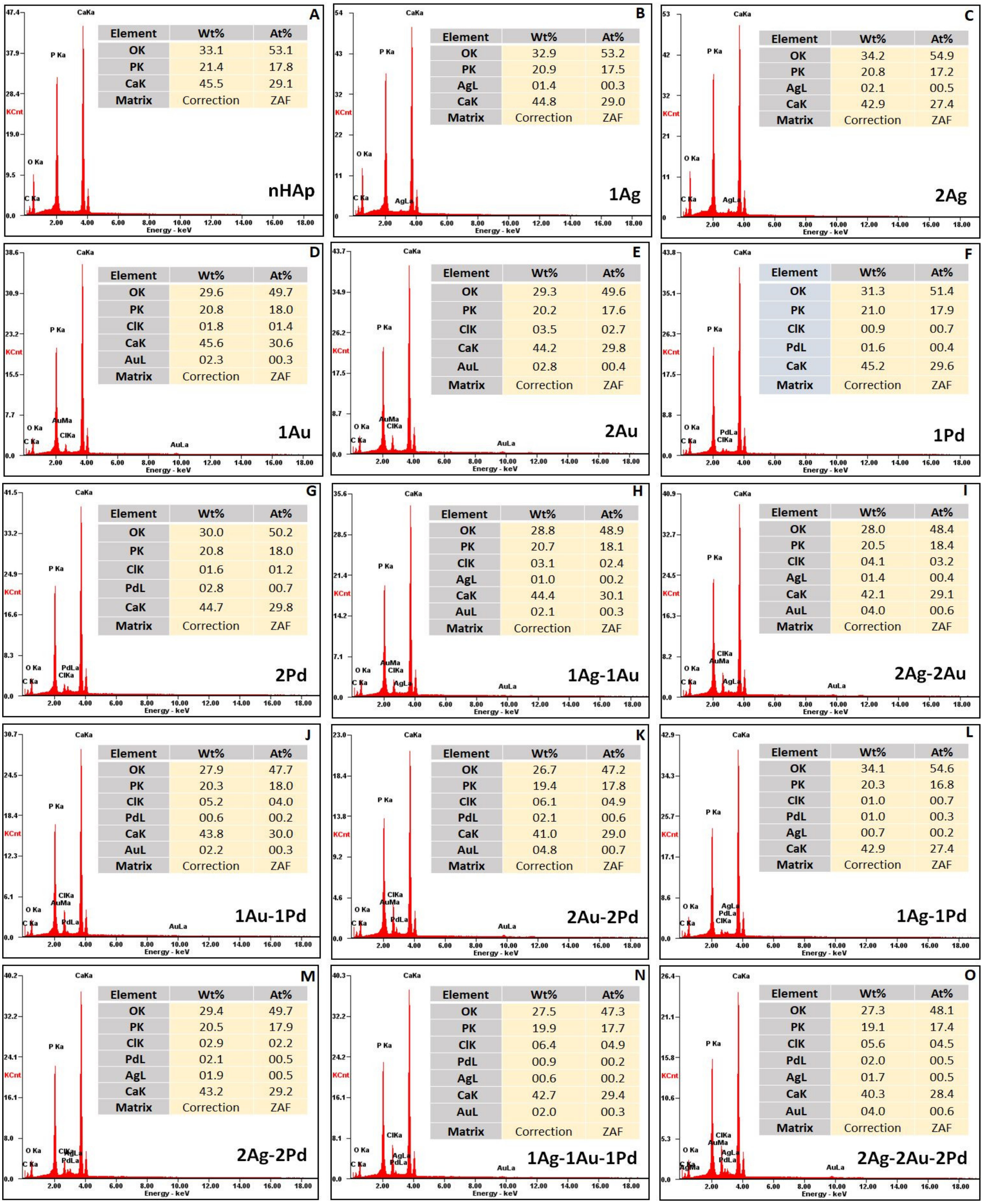

A (nHAp)

Element	Wt%	At%
OK	33.1	53.1
PK	21.4	17.8
CaK	45.5	29.1
Matrix	Correction	ZAF

B (1Ag)

Element	Wt%	At%
OK	32.9	53.2
PK	20.9	17.5
AgL	01.4	00.3
CaK	44.8	29.0
Matrix	Correction	ZAF

C (2Ag)

Element	Wt%	At%
OK	34.2	54.9
PK	20.8	17.2
AgL	02.1	00.5
CaK	42.9	27.4
Matrix	Correction	ZAF

D (1Au)

Element	Wt%	At%
OK	29.6	49.7
PK	20.8	18.0
ClK	01.8	01.4
CaK	45.6	30.6
AuL	02.3	00.3
Matrix	Correction	ZAF

E (2Au)

Element	Wt%	At%
OK	29.3	49.6
PK	20.2	17.6
ClK	03.5	02.7
CaK	44.2	29.8
AuL	02.8	00.4
Matrix	Correction	ZAF

F (1Pd)

Element	Wt%	At%
OK	31.3	51.4
PK	21.0	17.9
ClK	00.9	00.7
PdL	01.6	00.4
CaK	45.2	29.6
Matrix	Correction	ZAF

G (2Pd)

Element	Wt%	At%
OK	30.0	50.2
PK	20.8	18.0
ClK	01.6	01.2
PdL	02.8	00.7
CaK	44.7	29.8
Matrix	Correction	ZAF

H (1Ag-1Au)

Element	Wt%	At%
OK	28.8	48.9
PK	20.7	18.1
ClK	03.1	02.4
AgL	01.0	00.2
CaK	44.4	30.1
AuL	02.1	00.3
Matrix	Correction	ZAF

I (2Ag-2Au)

Element	Wt%	At%
OK	28.0	48.4
PK	20.5	18.4
ClK	04.1	03.2
AgL	01.4	00.4
CaK	42.1	29.1
AuL	04.0	00.6
Matrix	Correction	ZAF

J (1Au-1Pd)

Element	Wt%	At%
OK	27.9	47.7
PK	20.3	18.0
ClK	05.2	04.0
PdL	00.6	00.2
CaK	43.8	30.0
AuL	02.2	00.3
Matrix	Correction	ZAF

K (2Au-2Pd)

Element	Wt%	At%
OK	26.7	47.2
PK	19.4	17.8
ClK	06.1	04.9
PdL	02.1	00.6
CaK	41.0	29.0
AuL	04.8	00.7
Matrix	Correction	ZAF

L (1Ag-1Pd)

Element	Wt%	At%
OK	34.1	54.6
PK	20.3	16.8
ClK	01.0	00.7
PdL	01.0	00.3
AgL	00.7	00.2
CaK	42.9	27.4
Matrix	Correction	ZAF

M (2Ag-2Pd)

Element	Wt%	At%
OK	29.4	49.7
PK	20.5	17.9
ClK	02.9	02.2
PdL	02.1	00.5
AgL	01.9	00.5
CaK	43.2	29.2
Matrix	Correction	ZAF

N (1Ag-1Au-1Pd)

Element	Wt%	At%
OK	27.5	47.3
PK	19.9	17.7
ClK	06.4	04.9
PdL	00.9	00.2
AgL	00.6	00.2
CaK	42.7	29.4
AuL	02.0	00.3
Matrix	Correction	ZAF

O (2Ag-2Au-2Pd)

Element	Wt%	At%
OK	27.3	48.1
PK	19.1	17.4
ClK	05.6	04.5
PdL	02.0	00.5
AgL	01.7	00.5
CaK	40.3	28.4
AuL	04.0	00.6
Matrix	Correction	ZAF

Figure S1. EDS spectra of the nHAp (A) and nanoapatites doped (B-G), double-doped (H-M) and triple-doped (N,O) with Ag^{+}, Au^{+} and Pd^{2+} at a concentration of 1 $_{mol}$% and 2 $_{mol}$%, with the indication of Cl^{-} ions. The table inside contains quantitative measurement data. Sample names have been abbreviated, e.g. sample code OH-Cl-Ap: 1$_{mol}$% Ag^{+}, 1 $_{mol}$% Au^{+} is 1Ag-1Au.

Representative SEM images of the nHAp pellet and nanoapatites pellets doped with Ag^+, Au^+ and Pd^{2+} ions

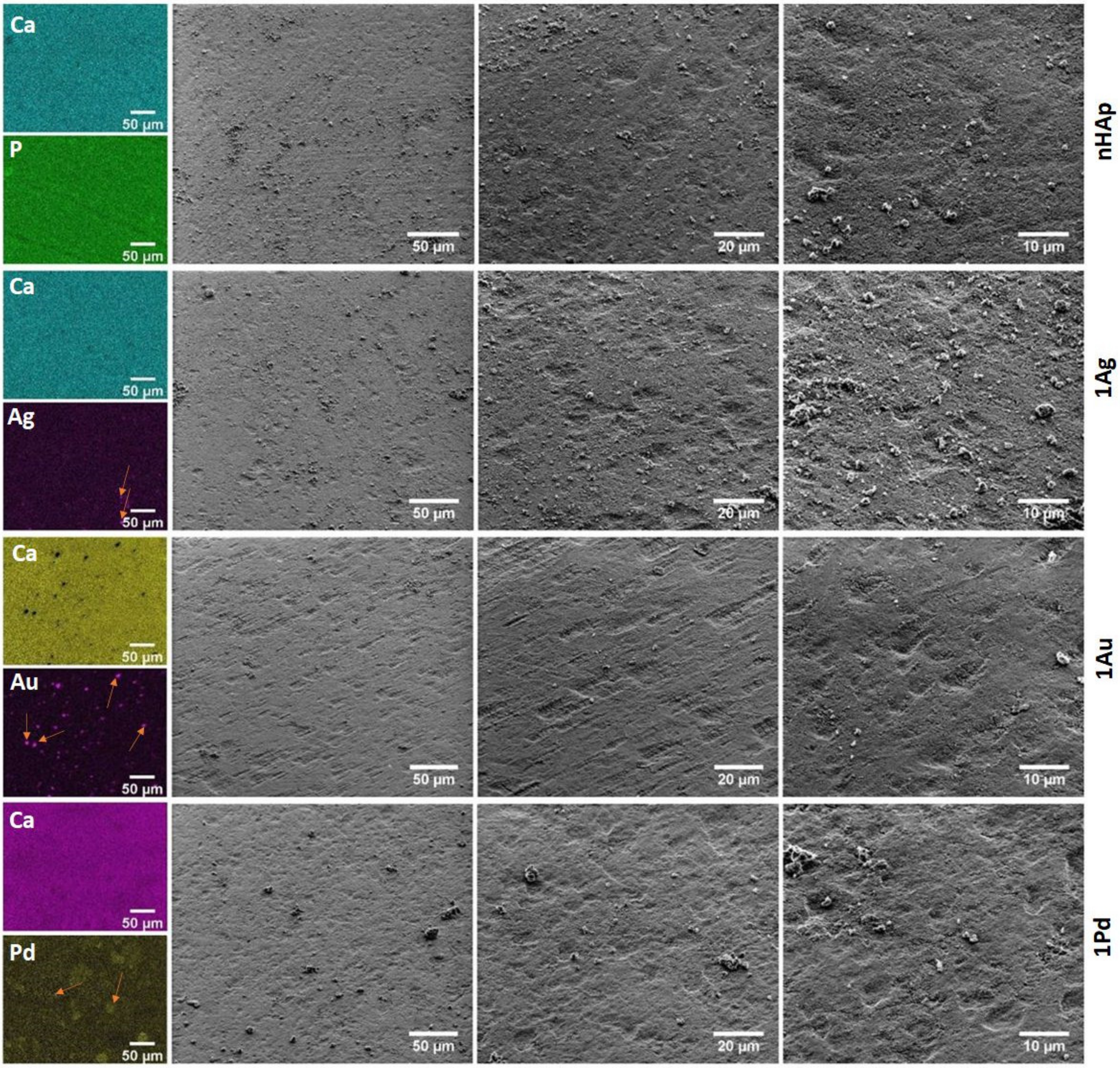

Figure S2. Representative SEM images (50 µm scale bar - 1000x magnification, 20 µm- 2500x, 10 µm -5000x) of the nHAp pellet and nanoapatites pellets doped with Ag^+, Au^+ and Pd^{2+} ions at a concentration of 1 $_{mol}$% together with EDS elemental maps. Arrows on the maps indicate metallic precipitates. Sample names have been abbreviated, e.g. sample code OH-Cl-Ap: 1 $_{mol}$% Au^+ is 1Au.

Adhesion of Balb/3T3 fibroblasts to surface tested nanoapatites and influence of the biofilm produced by E. faecalis VRE 200 and S. aureus MRSA P19 on the viability of Balb/3T3 fibroblasts

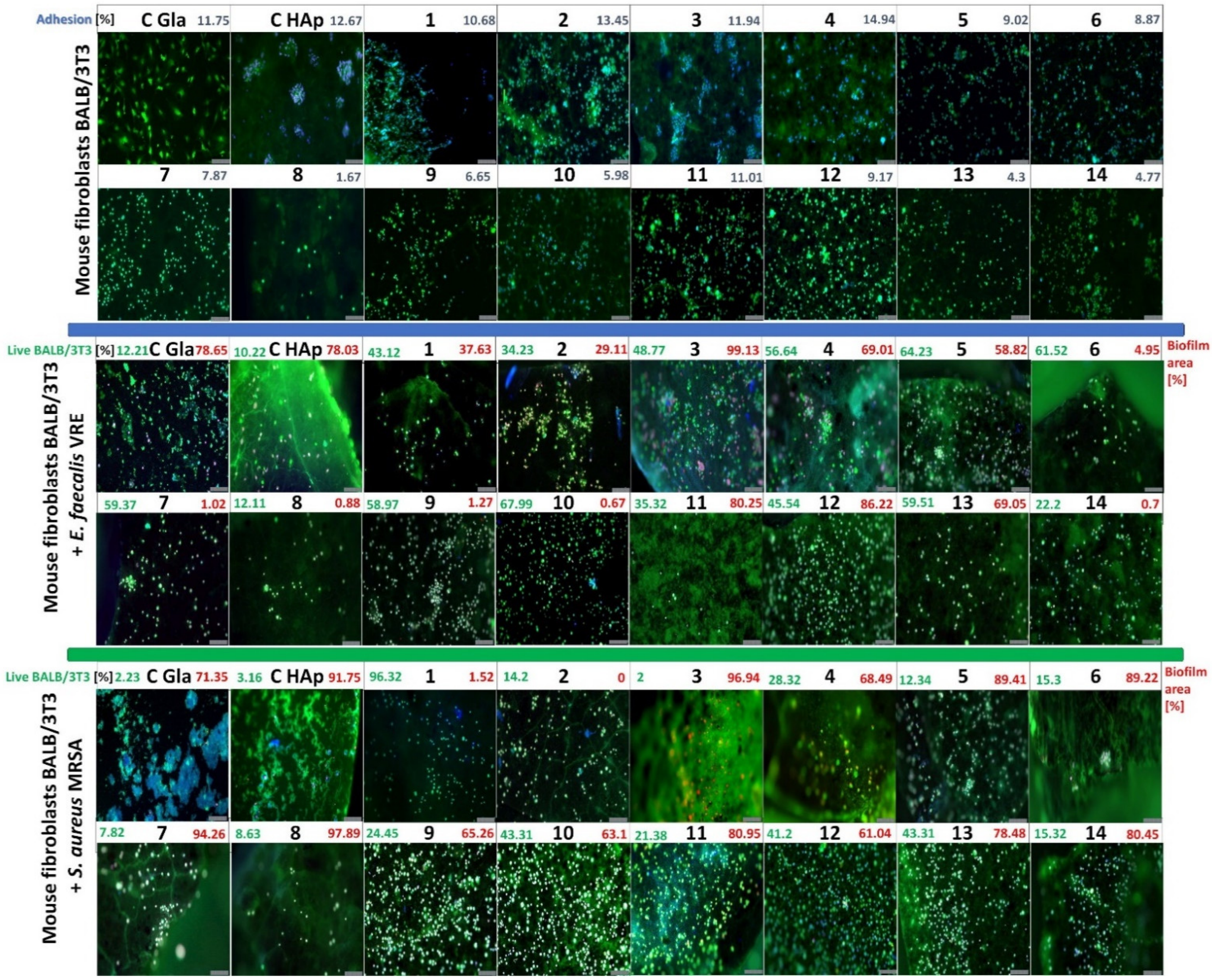

Figure S3. Images from the fluorescence microscopy visualisation presenting adhesion of Balb/3T3 mouse embryonic fibroblasts cells to the suface of tested biomaterials (top). Information above the photos: adhesion of fibroblasts [%] (right side) and influence of the bacterial biofilm produced by *E. faecalis* VRE 200 (in the middle) or *S. aureus* MRSA P19 (bottom) on the viability of fibroblast cells on tested nanoapatites dopped of nobile metals. Information above the photos: on the left side, the Balb/3T3 viability [%]; on the right side: biofilm area [%] on surfaces of nanoapatites discs. C HAp and C Gla were a pure nanohydroxyapatite and control glass, respectively, and constituted controls of experiments. Scale bars = 100 μm.
